# The Correlation Between Fear Avoidance Beliefs and Physical Activity in Unilateral Vestibulopathies

**DOI:** 10.1097/NPT.0000000000000499

**Published:** 2024-10-17

**Authors:** Lien Van Laer, Hanna M. Koppelaar-van Eijsden, Ann Hallemans, Vincent Van Rompaey, Tjard R. Schermer, Tjasse D. Bruintjes, Luc Vereeck

**Affiliations:** Department of Rehabilitation Sciences and Physiotherapy/Movant, Faculty of Medicine and Health Science, University of Antwerp, Antwerp, Belgium (L.V.L., A.H., and L.V.); Multidisciplinary Motor Centre Antwerp Department (M2OCEAN), University of Antwerp, Antwerp, Belgium (L.V.L., A.H., and L.V.); Apeldoorn Dizziness Centre Department, Gelre Hospitals, Apeldoorn, Gelderland, the Netherlands (H.M.K.E., T.R.S., and T.D.B); Department of Otorhinolaryngology, Leiden University Medical Center, Leiden, the Netherlands (H.M.K.E. and T.D.B.); Department of Otorhinolaryngology and Head & Neck Surgery, and Department of Translational Neurosciences, Faculty of Medicine and Health Sciences, University of Antwerp, Antwerp University Hospital, Antwerp, Belgium (V.V.R.); and Department of Primary and Community Care, Radboud Institute for Health Sciences, Radboud University Medical Center, Nijmegen, the Netherlands (T.R.S.).

**Keywords:** dizziness, fear avoidance, physical activity, unilateral vestibulopathy, vestibular rehabilitation

## Abstract

**Background and Purpose:**

In individuals with unilateral vestibulopathy (UVP), physical activity (PA) is recommended to stimulate central vestibular compensation. However, the presence of fear avoidance beliefs might negatively influence PA. The objectives of this study were to investigate the relationship between fear avoidance beliefs and PA and to compare PA levels between individuals with UVP in an acute/subacute vs chronic phase.

**Methods:**

In this cross-sectional study, PA was measured using a triaxial accelerometer. Fear avoidance beliefs were quantified using the Vestibular Activities Avoidance Instrument. The correlation between fear avoidance beliefs and PA was evaluated using regression analyses, with other potential influencing factors also considered.

**Results:**

A total of 102 participants were included. The average age was 56.1 (SD 15.2) years, and 57.8% were male. Participants with chronic UVP presented with shorter sedentary time (4,591 vs 5129 min/wk; *P* = 0.004), longer standing time (1443 vs 1165 min/wk; *P* = 0.025), higher vigorous PA (187 vs 107 min/wk; *P* = 0.005), and higher total PA (773 vs 623 min/wk; *P* = 0.003) compared to participants with acute/subacute UVP. In participants with acute/subacute UVP, variability in total PA was explained up to 54.7% by fear avoidance beliefs, etiology of the UVP, and gender (*R*^2^ = 0.547, *F*_4,45_ = 13.6, *P* < 0.001). In participants with chronic UVP, fear avoidance beliefs explained 4.1% of the variability in total PA (*R*^2^ = 0.041, *F*_1,49_ = 2.086, *P* = 0.155).

**Discussion and Conclusions:**

In acute/subacute UVP, assessing fear avoidance beliefs helps to understand physical inactivity. In chronic UVP, no significant association between fear avoidance beliefs and PA was observed.

**Video Abstract available:**

for more insights from the authors (see the video, Supplemental Digital Content, available at: http://links.lww.com/JNPT/A488).

## INTRODUCTION

The importance of physical activity (PA) and its beneficial effect on general health is well known.^1^ However, physical inactivity is common not only in healthy adults but also in individuals with vestibular disorders, such as unilateral vestibulopathy (UVP).^2^,^3^ It is routinely advised that individuals stay physically active to maintain their physical condition. In addition, in individuals with UVP, PA and exposure to movement can stimulate the process of central vestibular compensation.^4^,^5^ However, after UVP, head movements often provoke symptoms such as vertigo, due to an asymmetric response of the vestibular organs caused by UVP. Consequently, symptoms associated with movement may cause individuals with UVP to develop anxiety, which might result in movement restriction. This behavioral response is referred to as “fear avoidance.”^6^ Such a response to dizziness can lead to a maladaptation of the vestibular system and thus to chronic symptoms.^7^ Vestibular rehabilitation can help reduce this fear and thereby improve central vestibular compensation.^5^

Fear avoidance is measured by the Vestibular Activities Avoidance Instrument (VAAI).^6^ The literature has revealed that in individuals with UVP, fear avoidance is related to symptoms of depression and anxiety, reduced quality of life, and lower activity and participation levels.^8^,^9^ Kamo et al observed a negative relationship between PA and perceived handicap in individuals with dizziness.^10^ Furthermore, lower PA levels were found in individuals with chronic peripheral vestibulopathy compared to healthy controls.^11^

The reason for lower PA levels in individuals with UVP remains unclear. We hypothesize that individuals with UVP who have higher levels of fear avoidance are less physically active, which could influence vestibular and general recovery. This hypothesis was supported by previous research.^9^,^12^ Nevertheless, it is unclear whether the relationship between fear avoidance and PA is similar in individuals in the acute/subacute and chronic phases of UVP.^9^ Due to vestibular asymmetry during the acute/subacute UVP phase, individuals might have the tendency to avoid movements that potentially provoke symptoms. However, during the chronic UVP phase, it is expected that central vestibular compensation will lead to symptom-free movement. To summarize, our objectives were: (1) to investigate the correlation between fear avoidance beliefs and objectively measured PA in individuals with a diagnosis of UVP; (2) to investigate whether the level of PA differed between individuals with acute/subacute and chronic UVP; and (3) whether the relationship between PA and fear avoidance beliefs differed between individuals with acute/subacute UVP and chronic UVP.

## METHODS

### Ethical Considerations

The study was conducted in accordance with the Declaration of Helsinki and was approved by the ethics committee of the Antwerp University Hospital (registration no. 21/12/181) and Leiden Den Haag Delft (NL77986.058.21). All participants gave written informed consent before participating in the study. The study protocol was registered on clinicaltrials.gov (registration no. NCT04979598).

### Design, Setting, and Procedure

A cross-sectional study was performed between May 2021 and January 2023. Eligible individuals with UVP were recruited by a neurologist and/or ear, nose, and throat surgeon at different hospitals in Belgium (Antwerp University Hospital; Jessa Hospital, Hasselt; and Sint-Lievenspoort Rehabilitation Centre, Ghent) and in the Netherlands (Gelre Hospital). A member of the research staff contacted potential participants to inform them about the study and to obtain informed consent. Study participants completed a questionnaire and wore an activity logger for 1 week. At enrollment in the study, participants received the advice to be as physically active as possible from a medical doctor or the researchers and to start vestibular rehabilitation, as this is considered standard care after UVP.^5^ Vestibular rehabilitation was offered through a home exercise program or by referral to a primary care physical therapist with knowledge of vestibular rehabilitation for supervised sessions.

### Participants

Individuals diagnosed with UVP were included if they met the Barany Society diagnostic criteria for unilateral vestibular hypofunction, which concerns complaints of dizziness and/or balance problems, and verified vestibular function loss (ie, ipsilesional vestibulo-ocular reflex [VOR] gain < 0.70, a VOR gain side difference > 0.30, and/or a caloric side difference ≥ 25%).^3^ Other inclusion criteria were (1) adult age; (2) capacity to understand Dutch and provide written informed consent; and (3) willing and able to wear the activity logger for 1 week. Exclusion criteria were other vestibulopathies (eg, bilateral, central, benign paroxysmal positional vertigo [BPPV]) or (acute) neurological disorders.

### Primary Outcome Measures

Physical activity levels were quantified using a MOX1 logger (Maastricht Instruments), which is a waterproof triaxial accelerometer.^13^ Participants wore the logger on their upper-right leg, approximately 15 cm above the knee, for 1 week. The logger records raw acceleration data at a sampling rate of 25 Hz. The recordings of the PA were quantified and classified using IDEEQ 2.0 software.^13^ The logger estimates the time spent in different body positions (ie, sitting/lying and standing) and different levels of activities (low, moderate, and vigorous).^14^,^15^

Only data collected in the time between getting up in the morning and going to bed in the evening were used for the analysis. Total physical activity (TPA) was classified as the sum of low physical activity (LPA), moderate physical activity (MPA), and vigorous physical activity (VPA). The results were reported as the number of minutes spent at these activity levels per week (a 7-day period). A period of 1 week was chosen to compare the results with the World Health Organization guidelines.^14^ If measurements were made for more or less than 7 days, the data were interpolated or extrapolated to 1 week, meaning that the number of minutes spent on PA was divided by the number of days measured and then multiplied by 7. To be eligible for analysis, a minimum of 3 days of recording time was required.

Fear avoidance beliefs were objectified using the total score of the Dutch 9-item version of the VAAI.^6^,^16^ The questionnaire consists of 9 statements scored on a 7-point Likert scale regarding 3 categories: work, fear, and activity and participation. The higher the score (range 0-54), the higher the likelihood of the presence of fear avoidance beliefs. The VAAI has demonstrated excellent internal consistency (α = 0.91) and test-retest reliability (intraclass correlation coefficient (ICC) = 0.92), with a minimal detectable change of 8.9 points in individuals with a vestibular disorder.^12^,^16^

### Secondary Outcome Measures

Perceived handicap was measured by means of the Dutch language version of the Dizziness Handicap Inventory (DHI). It is a self-report questionnaire that quantifies the impact of dizziness and imbalance on daily life by measuring self-perceived handicap.^17^,^18^ It consists of 25 items, with the total score ranging from 0 to 100 points and a higher score indicating greater dysfunction.^19^ It is advised that the total score of the DHI be used.^20^ The DHI-Dutch language version has also been shown to be a reliable instrument with sufficient construct validity in individuals with vestibular disorders.^18^,^20^,^21^

Anxiety and depression were measured by means of the Hospital Anxiety and Depression Scale (HADS).^22^,^23^ The HADS consists of 14 items divided into two, 7-item subscales: anxiety (HADS-A) and depression (HADS-D). Higher scores indicate greater levels of anxiety or depression. In dizzy individuals, the HADS is considered an acceptable tool to assess general psychiatric distress in these 2 domains.^24^ The test-retest reliability of the total scale and the subscales were good in a Dutch population.^23^

Data on the following variables were also collected: demographic characteristics (age and gender); outcome of a vestibular function test (percentage of caloric asymmetry and VOR gain values); time since onset of the UVP; primary UVP diagnoses and etiology; and whether or not a participant had physical therapy at the time of the measurement. Vestibular function was tested by means of caloric testing: at Gelre Hospital using Vestlab (Otometrics); at Antwerp University Hospital using Kaloristar (Biomed); and at Jessa Hospital and the referring hospital to Sint-Lievenpoort using Aquastar (Difra) and/or the video Head Impulse Test (vHIT) (ICS-Impulse vHIT, Otometrics/Natus).

Participants were classified as being in an acute or subacute phase when the time since onset was within 3 months (time since onset < 2 weeks or between 2 weeks and 3 months, respectively).^5^ When the time since onset was 3 months or more, the phase of recovery was classified as chronic. The diagnoses were categorized into 3 types of etiology: inflammatory, iatrogenic, or other.

### Statistical Analysis

A convenience sample was collected without prior power analysis. Demographic and clinical characteristics of the participants were described using frequencies, means, and standard deviations depending on the nature of the data. Data on the continuous variables were checked visually for the presence of a normal distribution (Q-Q plots and bell-shape of the histograms), which is known as the Eyeball test.^25^ Differences in demographic and clinical characteristics between acute/subacute UVP and chronic UVP participants were assessed by means of the χ^2^ test or independent samples *t* test. For the continuous variables, effect sizes were reported using Cohen’s *d* with accompanying 95% confidence intervals where appropriate.^26^

The relationship between PA (dependent variable) and fear avoidance beliefs (VAAI score) (independent variable) was analyzed in the following steps. First, we analyzed the correlation between each of the different levels of PA (ie, LPA, MPA, VPA, and TPA) and the VAAI total score using Pearson correlation coefficients and univariate linear regression. The level of PA with the highest correlation was used to further assess the relationship between PA and the VAAI score. Second, to determine whether we could consider our study population with participants in different phases of recovery from UVP as a single sample, we explored whether there was a difference between the participants with acute/subacute UVP and those with chronic UVP. This was tested using a linear regression model, with PA as the dependent variable and the VAAI score as the independent variable. The variable “time since onset” (ie, “acute/subacute” or “chronic”) was added as a covariate. In addition, an interaction term (VAAI score × time since onset) was included in the regression model to determine whether the relation between PA and the VAAI was modified by the time since onset. The inclusion of interaction terms allowed the exploration of whether the association between the dependent and independent variables was modified by a third covariate. If this interaction was found to be statistically significant (indicating effect modification; ie, a different relation between PA and fear avoidance beliefs in acute/subacute UVP compared to chronic UVP participants), the groups would be further analyzed separately.

Third, to check for other confounding factors and possible interaction with the VAAI score, a series of linear regression models were applied to examine the following variables: gender, age, ipsilesional VOR gain, etiology, whether participants had physical therapy or not at the time of measurement, and the scores on the DHI, HADS-A, and HADS-D. If the regression coefficient of one of the confounders or interaction terms had a significance level below 0.05, it was taken into account in the final linear regression model.

Finally, a multivariable linear regression model was applied to assess the relationship between PA, VAAI score, and other statistically significant variables from the previous step.

To facilitate the interpretation of the model output and avoid multicollinearity between the covariates, continuous variables were centered around their means before entering them into the regression analysis.^27^ All analyses were performed using IBM Statistics SPSS 27 for Windows.

## RESULTS

### Participant Characteristics

A total of 102 participants with UVP were included, of which 51 participants were in the acute/subacute phase and 51 participants in the chronic phase (Table 1). Time since onset of the UVP was within 2 weeks (n = 35), between 2 weeks and 3 months (n = 16), 3 months to 2 years (n = 23), and over 2 years (n = 28). Participants’ characteristics are presented in Table 1. A number of acute/subacute UVP and chronic UVP participants received a home exercise program on vestibular rehabilitation without a referral for supervised physical therapy sessions (n = 30 and n = 5, respectively). The remaining participants were referred to a primary care physical therapist to perform supervised physical therapy sessions (21 participants with acute/subacute UVP and 46 participants with chronic UVP). At the moment of the MOX measurement, 17 of these participants with acute/subacute UVP and 11 with chronic UVP were actually undertaking supervised physical therapy sessions for their vestibular complaints (Table 1; *P* = 0.183).

**Table 1.** Participant Characteristics and Differences Between Participants With (Sub)Acute and Chronic UVP

	Total Group (n = 102)	(Sub)Acute Participants (n = 51)	Chronic Participants (n = 51)	P Value Difference Between Acute and Chronic Participants
Age, mean (SD), y	56.1 (15.2)	53.7 (16.1)	58.5 (14.0)	0.106^c^
Females, n (%)	43 (42.2)	21 (40.4)	22 (43.1)	0.841^d^
Supervised physiotherapy at the time of the measurement, n (%) yes	28 (27.5)	17 (33.3)	11 (21.6)	0.183^d^
UVP characteristics				
VOR gain ipsilesional,^a^ mean (SD)	0.60 (0.25)	0.53 (0.23)	0.67 (0.25)	**0.006^c^**
VOR gain contralesional,^a^ mean (SD)	0.95 (0.20)	0.97 (0.23)	0.92 (0.15)	0.223^c^
Caloric asymmetry (%)	57.7 (20.8)	67.2 (26.3)	53.6 (16.7)	**0.048^d^**
Affected side, n (%) left	55 (53.9)	26 (51.0)	29 (56.9)	0.551^d^
Primary diagnosis, n (%)				**<0.001^e^**
Vestibular neuritis	48 (47.1)	23 (45.1)	25 (49.0)	
Labyrinthitis	13 (12.7)	6 (11.8)	7 (13.7)	
Menière’s disease	5 (4.9)	0 (0)	5 (9.8)	
Idiopathic peripheral	9 (8.8)	0 (0)	9 (17.6)	
Benign recurrent vertigo	1 (1.0)	0 (0)	1 (2.0)	
Traumatic	1 (1.0)	1 (2.0)	0 (0)	
Resection vestibular schwannoma	18 (17.6)	16 (31.4)	2 (3.9)	
Gentamicin injection	3 (2.9)	2 (3.9)	1 (2.0)	
Other iatrogenic	4 (3.9)	3 (5.9)	1 (2.0)	
Etiology, n (%)				**<0.001^e^**
Inflammatory	61 (59.8)	29 (56.9)	32 (62.7)	
Iatrogenic	25 (24.5)	21 (41.2)	4 (7.8)	
Other^b^	16 (15.7)	1 (2.0)	15 (29.4)	

Abbreviations: n, number; SD, standard deviation; UVP, unilateral vestibulopathy; VOR, vestibulo-ocular reflex.

^a^ The VOR gain is the ratio of the speed of the corrective eye movement to the speed of the head during the video Head Impulse Test.

^b^ Other etiologies consisted of idiopathic peripheral vestibular disorders, recurrent vestibulopathies, Menière’s disease, and UVP with a traumatic cause.

^c^ Results on the independent samples’ *t* test.

^d^ Results on the chi square test.

^e^ Results on the Fischer’s exact test.

Bold font type was used to indicate a statistically significant difference between acute and chronic participants.

### PA and Patient-Reported Outcome Measures

Table 2 shows data on the MOX measurements and the questionnaires. In 67 participants (65.7%), PA was measured over a period of 7 days. The MOX1 outcome parameters revealed that participants with acute/subacute UVP had a statistically significant higher sedentary time, lower standing time, lower VPA, and lower TPA compared to participants with chronic UVP. Moreover, we found statistically significant higher scores on the VAAI and lower scores on the DHI in participants with acute/subacute UVP compared to participants with chronic UVP.

**Table 2.** Outcome of the MOX Loggers and Questionnaires

	Total Group (n = 102)	(Sub)Acute Participants (n = 51)	Chronic Participants (n = 51)	*P* Value and Effect Size (95% CI) for Difference Between Participants With (Sub)Acute and Chronic UVP
Duration MOX measurement (d), mean (SD)	6.6 (0.9)	6.6 (0.9)	6.6 (0.8)	*P* = 1.000; Cohen’s *d* = 0.000 (−0.392; 0.392)^a^
MOX measurement of 7 d, n (%)	67 (65.7)	38 (74.5)	29 (56.9)	***P* = 0.009^b^**
MOX measurement of 3 d	1 (1)	0 (0)	1 (2)	
MOX measurement of 3.5 d	1 (1)	1 (2)	0 (0)	
MOX measurement of 4 d	2 (2)	2 (3.9)	0 (0)	
MOX measurement of 4.5 d	3 (2.9)	2 (3.9)	1 (2)	
MOX measurement of 5 d	3 (2.9)	0 (0)	3 (5.9)	
MOX measurement of 5.5 d	3 (2.9)	3 (5.9)	0 (0)	
MOX measurement of 6 d	8 (7.8)	1 (2.0)	7 (13.7)	
MOX measurement of 6.5 d	12 (11.8)	4 (7.8)	8 (15.7)	
MOX measurement of 7.5 d	1 (1)	0 (0)	1 (2)	
MOX measurement of 8.0 d	1 (1)	0 (0)	1 (2)	
MOX outcome (min/wk), mean (SD)				
Sitting/lying	4860 (944)	5129 (905)	4591 (913)	***P* = 0.004; Cohen’s *d* = 0.592 (0.194; 0.988)^a^**
Standing	1304 (631)	1165 (699)	1443 (525)	***P* = 0.025; Cohen’s *d* = −0.449 (−0.841; −0.055)^a^**
Low physical activity	87 (31)	81 (37)	93 (35)	*P* = 0.079; Cohen’s *d* = −0.351 (−0.742; 0.041)^a^
Moderate physical activity	465 (248)	438 (277)	493 (214)	*P* = 0.251; Cohen’s *d* = −0.229 (−0.618; 0.161)^a^
Vigorous physical activity	147 (146)	107 (130)	187 (152)	***P* = 0.005; Cohen’s *d* = −0.565 (−0.960; −0.168)^a^**
Total physical activity	698 (357)	623 (391)	773 (304)	***P* = 0.033; Cohen’s *d* = −0.428 (−0.820; −0.035)^a^**
Questionnaire scores, mean (SD)				
VAAI	31.8 (11.4)	34.4 (11.2)	29.2 (11.1)	***P* = 0.021; Cohen’s *d* = 0.464 (0.070; 0.857)^a^**
DHI	44.6 (19.2)	40.2 (18.4)	49.1 (19.2)	***P* = 0.018; Cohen’s *d* = −0.475 (−0.868; −0.081)^a^**
HADS-A	6.0 (4.1)	6.2 (4.3)	5.8 (3.8)	*P* = 0.708; Cohen’s *d* = 0.077 (−0.325; 0.479)^a^
HADS-D	5.1 (3.6)	4.6 (3.5)	5.6 (3.5)	*P* = 0.160; Cohen’s *d* = −0.291 (−0.694; 0.114)^a^

Abbreviations: DHI, Dizziness Handicap Inventory; HADS-A, Hospital Anxiety and Depression Scale—Subscale Anxiety; HADS-D, Hospital Anxiety and Depression Scale—Subscale Depression; SD, standard deviation; UVP, unilateral vestibulopathy; VAAI, Vestibular Activities Avoidance Instrument.

^a^ Results on independent samples *t* test.

^b^ Results on Fischer’s exact test.

Bold font type was used to indicate a statistically significant difference between acute and chronic participants.

### Relationship Between Fear Avoidance Beliefs and PA

**Figure 1.**
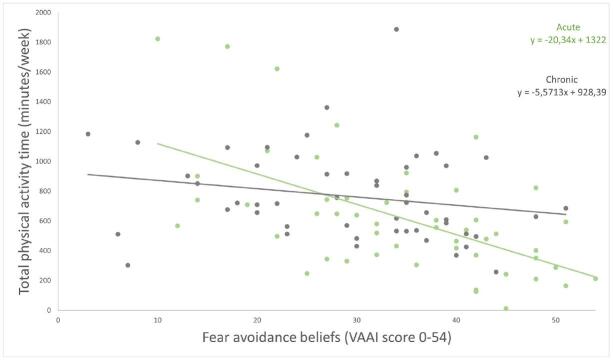
The relationship between fear avoidance beliefs and physical activity compared between participants with an acute and chronic UVP. UVP, unilateral vestibulopathy; VAAI, Vestibular Activities Avoidance Instrument. The relation between fear avoidance beliefs and physical activity significantly differed between participants the (sub)acute and chronic phase of the UVP (*P* = 0.009). This figure is available in color online (www.jnpt.org).

First, the initial correlation and regression analyses identified TPA as the PA outcome with the strongest correlation (*r* = −0.441)—albeit moderate in strength^28^—to fear avoidance beliefs (Table 3). Therefore, TPA was used as the dependent variable in the regression analyses. Subsequently, when checking for the influence of “time since onset,” the interaction term was found to be statistically significant (*P* = 0.009), meaning there was a different relationship between the VAAI and TPA for participants with acute/subacute UVP and those with chronic UVP (Figure 1). Therefore, further analysis of the relation between the VAAI and TPA was performed separately for the subgroups of acute/subacute UVP and chronic UVP participants.

**Table 3.** Correlation and Univariable Linear Regression Analysis Results With Physical Activity Parameters as the Dependent Variable and VAAI Score as the Independent Variable

Dependent Variables (min/wk)	*r*	Strength of Correlation	*P* Value of the Correlation Coefficient *r*	*R*^2^	Intercept	*Β*	*P* Value of the Regression Coefficient *B*
Sitting/lying	0.220	Weak	**0.026**	0.048	4280	18	**0.026**
Standing	−0.375	Weak	**<0.001**	0.140	1963	−21	**< 0.001**
Low physical activity	−0.375	Weak	**<0.001**	0.141	125	−1	**< 0.001**
Moderate physical activity	−0.359	Weak	**<0.001**	0.129	714	−8	**< 0.001**
Vigorous physical activity	−0.375	Weak	**<0.001**	0.140	299	−5	**< 0.001**
Total physical activity	−0.441	Moderate	**<0.001**	0.194	1136	−14	**< 0.001**

Abbreviations: *B*, regression coefficient of the regression equation; *r*, Pearson correlation coefficient; *R*^2^, coefficient of determination in regression; VAAI, Vestibular Activities Avoidance Instrument.

Bold font type was used to indicate a statistically significant result (*P* < 0.05).

#### Participants With Acute/Subacute Unilateral Vestibulopathy

The univariate linear regression models showed that in acute/subacute UVP participants, in addition to the total VAAI score (*P* < 0.001), also 3 other variables were significant explanatory factors for TPA: etiology (*P* < 0.001), gender (*P* < 0.001), and the interaction term between gender and the VAAI score (*P* = 0.031). The other covariates were not statistically significant.

**Figure 2.**
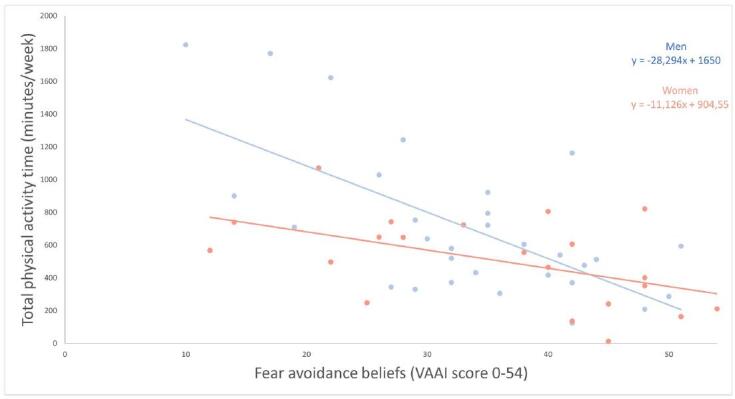
The relation between fear avoidance beliefs and physical activity compared between women and men in participants with an acute UVP. VAAI, Vestibular Activities Avoidance Instrument. The relation between fear avoidance beliefs and physical activity significantly differed between women and men in participants with an acute UVP (*P* = 0.031). This figure is available in color online (www.jnpt.org).

When comparing TPA between the inflammatory and iatrogenic etiologies, a higher number of TPA minutes was found in the inflammatory etiologies (776 ± 402 min/wk) compared to the iatrogenic etiologies (404 ± 241 min/wk) (*P* < 0.001). The relationship between the VAAI and TPA was stronger in men compared to women (*P* = 0.031) (Figure 2). However, the difference between men and women in TPA time was not statistically significant, at 704 ± 443 and 508 ± 272 min/wk (*P* = 0.077), respectively.

Finally, after combining all significant factors into 1 final multivariable regression model for the participants with acute/subacute UVP, 54.7% of the variability in TPA time was explained by VAAI score, etiology, gender, and the gender × VAAI interaction term (Table 4).

**Table 4.** Final Multivariate Linear Regression Models for the Acute and Chronic UVP Subgroups With Total Physical Activity (min/wk) as the Dependent Variable

Regression Model in Participants With (Sub)Acute UVP a			
**VAAI as Independent Variable and Covariates**	***B* (95% Confidence Intervals)**	***F*_1,49_**	**Level of Significance of the *F*-Value**
Fear avoidance beliefs (VAAI)	−7.3 (−17.6; 3.010)^e^	19.1	*P* < 0.001^f^
Etiology^c^	−245.7 (−416.0; −75.4)	8.4	*P* = 0.006^f^
Gender^d^	203.1 (38.0; 368.3)	6.1	*P* = 0.017^f^
Interaction gender and fear avoidance beliefs	−18.1 (−32.2; −3.9)	6.6	*P* = 0.013^f^
**Regression Model in Participants With Chronic UVP^b^**			
**Independent Variables**	***B* (95% Confidence Intervals)**	***F*_1,49_**	**Level of Significance of the *F*-Value**
Fear avoidance beliefs (VAAI)	−5.5 (−13.2; 2.2)	2.086	*P* = 0.155

Abbreviations: B, regression coefficient of the regression equation; F, ratio of the mean regression sum of squares divided by the mean error sum of squares; UVP, unilateral vestibulopathy; VAAI, Vestibular Activities Avoidance Instrument; x,y, degrees of freedom.

^a^ The regression model in the participants with (sub)acute UVP had an *R*^2^ value of 0.547, *F*_4,45_ = 13.6 (*P* < 0.001).

^b^ The regression model in the participants with chronic UVP had an *R*^2^ value of 0.041, *F*_1,49_ = 2.086 (*P* = 0.155).

^c^ For the covariate Etiology, the inflammatory etiology group was used as the reference category.

^d^ For the covariate Gender, the female gender was used as the reference category.

^e^ Note that the 95% confidence interval for the VAAI includes 0 as this B-coefficient is calculated for both men and women, leading to a not statistically significant value. However, based on the *F*-value, the VAAI was identified as the strongest individual explanatory factor for total physical activity (*P* < 0.001).

^f^ Significant result (*P* < 0.05).

#### Participants With Chronic Unilateral Vestibulopathy

For the participants with chronic UVP, no other significant variables were identified. Therefore, the final regression model for the participants with chronic UVP only consisted of the VAAI score, which explained 4.1% of the variability in TPA time (Table 4).

## DISCUSSION

### Summary of the Results and Interpretation

The objective of this study was to investigate the correlation between fear avoidance beliefs and PA in individuals with UVP. In acute/subacute UVP participants, fear avoidance was identified as the strongest explanatory factor for TPA. Our results suggest that fear avoidance beliefs partially explain why individuals in the acute/subacute phase are less physically active. Hence, individuals with acute/subacute UVP with elevated scores on the VAAI, indicating the presence of fear avoidance beliefs, might be at higher risk of physical inactivity.

Vestibular rehabilitation is highly recommended for individuals with UVP.^5^ In addition, assessing fear avoidance beliefs, monitoring PA, or offering additional interventions such as cognitive behavioral therapy or a walking program to promote PA and address avoidance beliefs should be considered.^29^-^32^ In addition to fear avoidance beliefs, etiology and gender were also identified as influencing factors for TPA in participants with acute/subacute UVP. In this group, participants with an iatrogenic etiology for their UVP had lower PA levels compared to those with inflammatory etiologies. The iatrogenic causes mainly consisted of resections of a vestibular schwannoma using the retrosigmoid approach.^33^ After this surgery, initially caution with head movement is advised. Moreover, after vestibular schwannoma surgery, the possible presence of complications, such as postoperative headaches, might also negatively influence PA.^34^,^35^ As a consequence, both the time needed to recover from the surgical intervention itself and the presence of complications could potentially decrease PA levels. Unfortunately, in this study, the presence of complications was not systematically monitored.

Regarding gender, a stronger correlation between fear avoidance and PA was observed in male participants compared to female participants. Although we were unable to find statistically significant differences in TPA time between men and women, there were 6 male high performers—with a TPA time > 1000 min/wk—compared to only 1 female high performer (Figure 2). Of these male high performers, 5 presented with a VAAI score below 30, probably contributing to a stronger relation between fear avoidance and PA in men.

We found no significant associations between fear avoidance and PA in participants with chronic UVP, whereas other studies have found conflicting evidence regarding the relation between PA and dizziness in this group.^10^,^36^

Furthermore, our results showed remarkably lower figures for TPA time in participants with chronic UVP compared to other studies that have measured and analyzed PA in a similar way^10^,^36^: approximately 111 min/d in our study and 360 to 392 min/d in previous research.^10^,^36^ The lower figures in our study might be explained by longer symptom duration, a more specific population (eg, exclusion of BPPV), and a higher perceived handicap (DHI = 49.1 ± 19.2) compared to the other studies (36.6 ± 23.8 and 41.3 ± 21.3).^10^,^36^ Although our participants tended to spend less time being physically active compared to other individuals with chronic vestibulopathy, on average, the World Health Organization guidelines regarding moderate and vigorous PA were met.^14^ All participants included in this study were advised to be physically active. This might explain why, in the majority of the participants, appropriate PA levels were reached and why—due to the low numbers of participants who were physically inactive—it was challenging to explain the variability in PA. In individuals with UVP, we advise that the presence of fear avoidance beliefs and PA during the acute/subacute phase be routinely assessed, especially in those with an iatrogenic etiology. Our results suggest a correlation between fear avoidance beliefs and limited PA, potentially influencing the healing process. In people with chronic UVP, PA levels are more difficult to explain. More research in this population is needed to explore alternatives for measuring PA (eg, intensity, location of accelerometer) and investigate whether other factors are involved.

### Strengths and Limitations

One strength of this study is that we measured PA objectively over a period of at least 3 days. In addition to the objective PA measures, we were able to document various relevant outcome measures, such as the presence of psychological factors. Furthermore, we attempted to avoid heterogeneity as much as possible by limiting the population of interest to individuals with UVP, following the diagnostic criteria of the Barany Society.^3^

There were also some limitations to this study. Based on suggestions from previous research^9^ and the fact that the relationship between PA and fear avoidance beliefs differed between the acute/subacute UVP and chronic UVP participants, we analyzed the data separately for both groups. By setting a cutoff point of 3 months, participants were artificially classified into 2 different groups. Nevertheless, this 3-month period is often used in the literature to mark the transition from the acute/subacute phase to the chronic phase.^5^,^37^ However, the relation between PA and fear avoidance beliefs might also differ between acute and subacute participants. Therefore, similar studies with 3 different groups (acute, subacute, and chronic phases) might lead to additional insights. Moreover, instead of using these 3 categories, another possibility might be to process and analyze time since onset as a continuous variable. We were not able to do so in this study as time since onset was collected as a categorical variable.

Another potential limitation is that the PA data were extrapolated to 1 week in 33 cases. Nevertheless, in 26 out of the 33 extrapolated measurements, at least 5 complete days of measurements were available, leading to a limited influence of the extrapolation. However, to our knowledge, no literature is available on the reliability of extrapolating activity data. In addition, upon enrollment in the study, participants were advised to stay physically active and to start vestibular rehabilitation through a home exercise program or referral to an external physical therapist. Nevertheless, at the moment of the measurement, only 28 participants were actually undertaking supervised physical therapy sessions. As supervised vestibular rehabilitation is highly recommended, we advise that all individuals with UVP be systematically referred to physical therapists.^5^

### CONCLUSIONS

The correlation between fear avoidance beliefs and PA differed in individuals with acute/subacute UVP and those in the chronic UVP phase. In participants with acute/subacute UVP, a reduction in PA could be explained up to 54.7% by fear avoidance beliefs, etiology of the UVP, and gender. Therefore, assessing fear avoidance beliefs will assist in identifying physical inactivity in individuals with acute/subacute UVP. In chronic UVP, no significant explanatory factors for PA were identified, and no significant association between fear avoidance beliefs and PA was observed. More research is needed on the predictive value of fear avoidance beliefs in relation to physical activity in individuals with chronic UVP.
